# The updated incidences and mortalities of major cancers in China, 2011

**DOI:** 10.1186/s40880-015-0042-6

**Published:** 2015-09-14

**Authors:** Wanqing Chen, Rongshou Zheng, Hongmei Zeng, Siwei Zhang

**Affiliations:** National Office for Cancer Prevention and Control, National Cancer Center, Beijing, 100021 P. R. China

**Keywords:** Cancer registry, Incidence, Mortality, Epidemiology, China

## Abstract

**Introduction:**

The National Central Cancer Registry (NCCR) of China collected population-based cancer registration data from all cancer registries in China. This study aimed to compile national cancer incidences and mortalities in 2011 and estimate cancer incident new cases and cancer deaths.

**Methods:**

In 2014, there were 234 cancer registries that submitted records of new cancer cases and cancer deaths that occurred in 2011 to the NCCR. All datasets were evaluated based on the criteria of data quality of the NCCR. The data of 177 registries was of sufficient quality and was compiled to evaluate cancer statistics in 2011. The pooled data were stratified by area, sex, age group, and cancer type. Cancer incident cases and deaths were estimated using age-standardized rates (ASR) and the Chinese population. All incidences and mortalities were age-standardized to the 2000 Chinese standard population and Segi’s population.

**Results:**

The estimates of new cancer incident cases and cancer deaths were 3,372,175 and 2,113,048 in 2011, respectively. The crude incidence was 250.28/1,00,000 (277.77/1,00,000 for males and 221.37/1,00,000 for females). The ASRs of incidence by the Chinese standard population (ASRIC) and by the world standard population (ASRIW) were 186.34/1,00,000 and 182.76/1,00,000, respectively, with a cumulative incidence (0–74 years old) of 21.20%. Cancers of the lung, female breast, stomach, liver, colorectum, esophagus, cervix, uterus, prostate, and ovary were the most common cancers, accounting for approximately 75% of all new cancer cases. Lung, liver, gastric, esophageal, colorectal, female breast, pancreatic, brain, and cervical cancers and leukemia were the leading causes of cancer death, accounting for approximately 80% of all cancer deaths. Cancer incidence, mortality, and spectrum were all different between urban and rural areas and between males and females.

**Conclusions:**

The population covered by the cancer registries greatly increased from 2010 to 2011. The data quality and representativeness of cancer registries have gradually improved. Cancer registries have an irreplaceable role in research on cancer prevention and control. The disease burden of cancer is increasing, and the health department must implement effective measures to contain the increased cancer burden in China.

## Introduction

Cancer is a major public health issue in China. It is the second leading cause of death, and its incidence and mortality continue to increase [1–3]. In 2008, the National Cancer Registration Program was launched by the Ministry of Health of China. Since then, the National Central Cancer Registry (NCCR) of China annually publishes cancer statistics based on the data reported by the cancer registries [4]. In this study, we analyzed the cancer incidence and mortality in cancer registration areas in 2011 and estimated the national numbers of new cancer cases and deaths to provide an overview of the current cancer statistics using data collected from local registries. The updated cancer burden results can be broadly used by the government, researchers, and clinicians for policy-making and research.

## Methods

### Data collection

A total of 234 cancer registries, located in 32 provinces, autonomous regions, and municipalities directly under the central government, submitted records of cancer new cases and deaths in 2011 to the NCCR. All data were evaluated based on the criteria of data quality of the NCCR. A total of 177 registries with qualified data were included in the final database for cancer statistics analysis in 2011. The registries identified new cancer cases from all hospitals, community health centers, medical insurance databases, and death registries (for cases only identified by death certification). The registries obtained information on cancer deaths from the death surveillance system, which collects death information from hospitals and the Civil Administration Bureau with available cremation records. Population information was collected from local statistical bureaus or household registry departments in local public security bureaus.

### Data quality control

The proportion of cases with morphologic verification (MV%), percentage of cancer cases identified by death certification only (DCO%), mortality to incidence (M/I) ratio, percentage of uncertified cancers (UB%), and percentage of cancers with undefined or unknown primary sites (secondary) (O&U%) were used to evaluate the completeness, validity, and reliability of the cancer statistics.

### Statistical analysis

Pooled data were stratified by area (urban and rural), sex (males and females), age group (0, 1–4, 5–9, …, 80–84, 85 years old and above), and cancer type. Cancer incident cases and deaths were estimated using age-specific rates, which were stratified by area, region (eastern, middle, western), cancer type, sex, and age. The 10 most common cancers in different groups were determined, and their proportions and cumulative rates were also calculated. The Chinese population census in 2000 and Segi’s population were used to determine age-standardized rates (ASRs) of incidence and mortality.

## Results

### Data sources

The 177 included cancer registries (77 in urban areas and 100 in rural areas) covered a total of 175,310,169 persons (98,341,507 in urban areas and 76,968,662 in rural areas). The overall indicators of MV%, DCO%, and M/I ratio were 70.14%, 2.44%, and 0.63, respectively. They were 72.92%, 2.17%, and 0.61 in urban registries and 65.34%, 2.90%, and 0.67 in rural registries, respectively.

### Incidences of all cancers

In 2011, 3,372,175 new cancer cases were estimated. The crude incidence in the Chinese cancer registration areas was 250.28/1,00,000 (277.77/1,00,000 for males and 221.37/1,00,000 for females), and the ASRs of incidence by the Chinese standard population (ASRIC) and by the world standard population (ASRIW) were 186.34/1,00,000 and 182.76/1,00,000, respectively, with a cumulative incidence (0–74 years old) of 21.20%. The crude incidence and ASRIC were 261.38/1,00,000 and 189.89/1,00,000 in urban areas and 238.60/1,00,000 and 182.10/1,00,000 in rural areas, respectively (Table 1). Cancer incidence was relatively lower in persons before age 39, increased dramatically after age 40, peaked after age 80, and then slightly decreased after age 85. This pattern was similar between urban and rural areas.

**Table 1:** Cancer incidence in China in 2011

Areas	Sex	Cancer cases	Crude incidence (1/10^5^)	ASRIC (1/10^5^)	ASRIW (1/10^5^)	Cumulative rate (%)
All	Both	3,372,175	250.28	186.34	182.76	21.20
	Male	1,918,533	277.77	213.66	212.18	25.02
	Female	1,453,642	221.37	161.47	155.84	17.43
Urban	Both	1,805,624	261.38	189.89	185.75	21.28
	Male	993,675	281.81	211.88	210.10	24.52
	Female	811,949	240.09	170.79	164.35	18.22
Rural	Both	1,566,551	238.60	182.10	179.24	21.08
	Male	924,858	273.57	215.54	214.38	25.55
	Female	641,693	201.49	150.57	146.02	16.51

*ASRIC* age-standardized rate of incidence by the Chinese standard population in 2000, *ASRIW* age-standardized rate of incidence by the Segi’s world standard population.

### Mortalities of all cancers

It was estimated that 2,113,048 persons died of cancer in 2011. The crude mortality in Chinese cancer registration areas was 156.83/1,00,000 (194.88/1,00,000 for males and 116.81/1,00,000 for females), and the ASRs of mortality by the Chinese standard population (ASRMC) and by the world standard population (ASRMW) were 112.88/1,00,000 and 111.82/1,00,000, respectively, with a cumulative incidence of 12.69%. The crude mortality and ASRMC were 154.37/1,00,000 and 108.20/1,00,000 in urban areas and 159.42/1,00,000 and 117.97/1,00,000 in rural areas, respectively (Table 2). The cancer mortality was relatively lower in persons before age 45 and then dramatically increased, reaching its peak after age 85. The mortality in rural areas was the highest in the age 80–84 group. The age-specific mortality of males was lower in urban areas than in rural areas in most age groups before age 80.

**Table 2:** Cancer mortality in China in 2011

Areas	Sex	Deaths	Crude mortality (1/10^5^)	ASRMC (1/10^5^)	ASRMW (1/10^5^)	Cumulative rate (%)
All	Both	2,113,048	156.83	112.88	111.82	12.69
	Male	1,345,998	194.88	148.28	147.44	16.72
	Female	767,050	116.81	79.42	78.31	8.67
Urban	Both	1,066,408	154.37	108.20	107.14	11.79
	Male	671,063	190.31	141.00	140.36	15.53
	Female	395,345	116.90	77.42	76.14	8.15
Rural	Both	1,046,640	159.42	117.97	116.84	13.65
	Male	674,935	199.64	156.03	154.83	17.98
	Female	371,705	116.71	81.66	80.71	9.22

*ASRMC* age-standardized rate of mortality by the Chinese standard population in 2000, *ASRMW* age-standardized rate of mortality by the Segi’s world standard population.

### The 10 most common cancers in China

**Figure 1:**
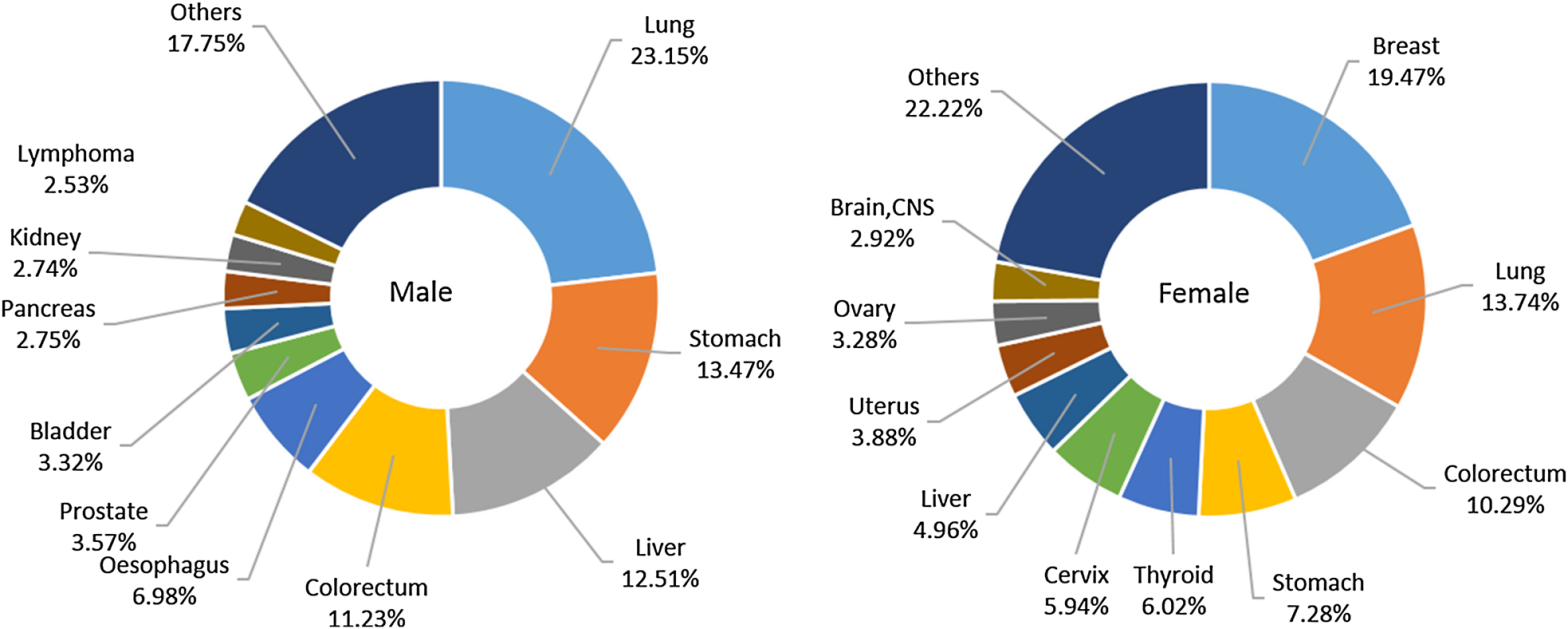
Ten cancers with the highest incidences in urban areas of China, 2011.

**Figure 2:**
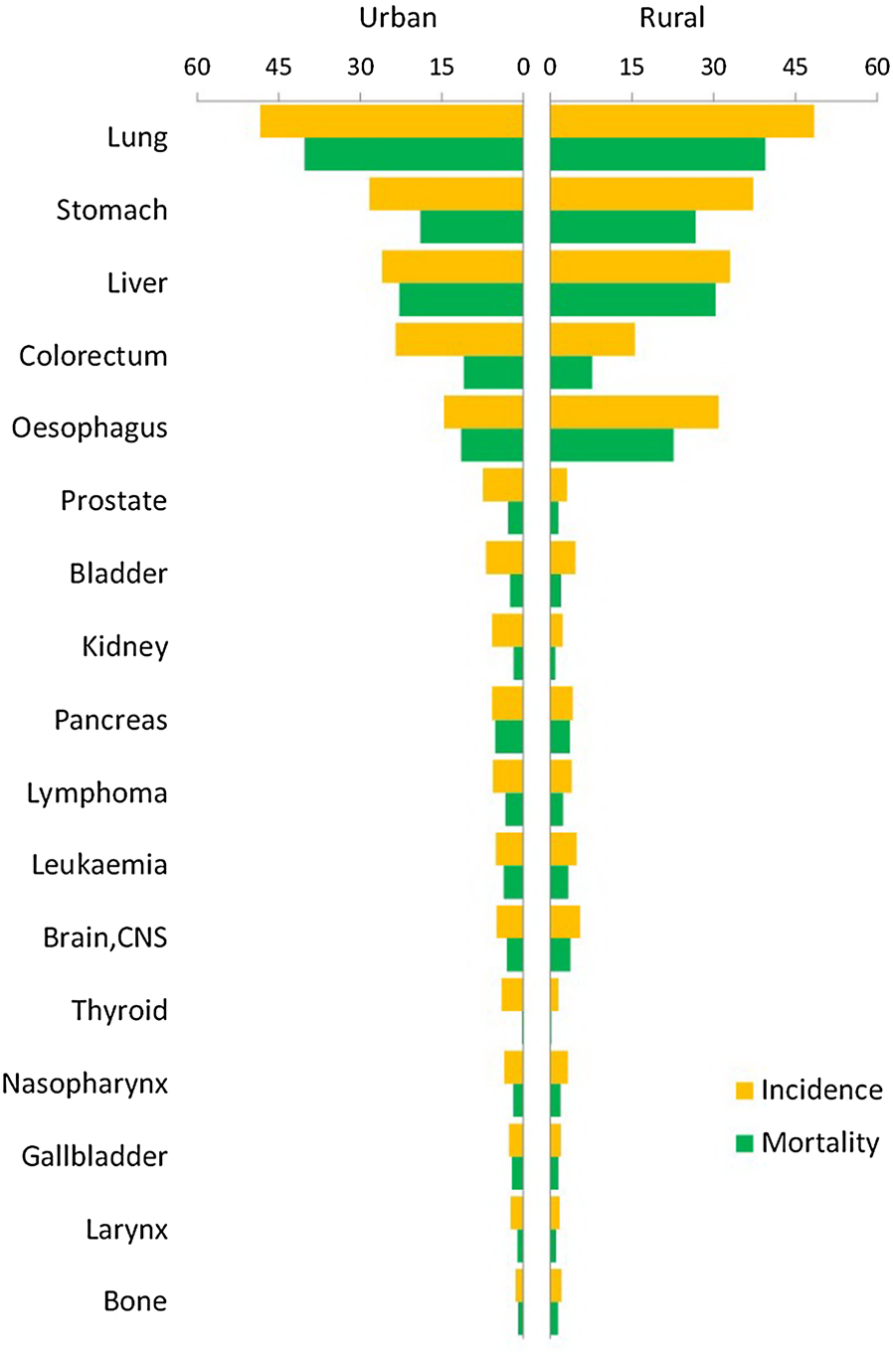
Cancer incidences and mortalities in urban and rural areas for males in China, 2011 (1/1,00,000).

**Figure 3:**
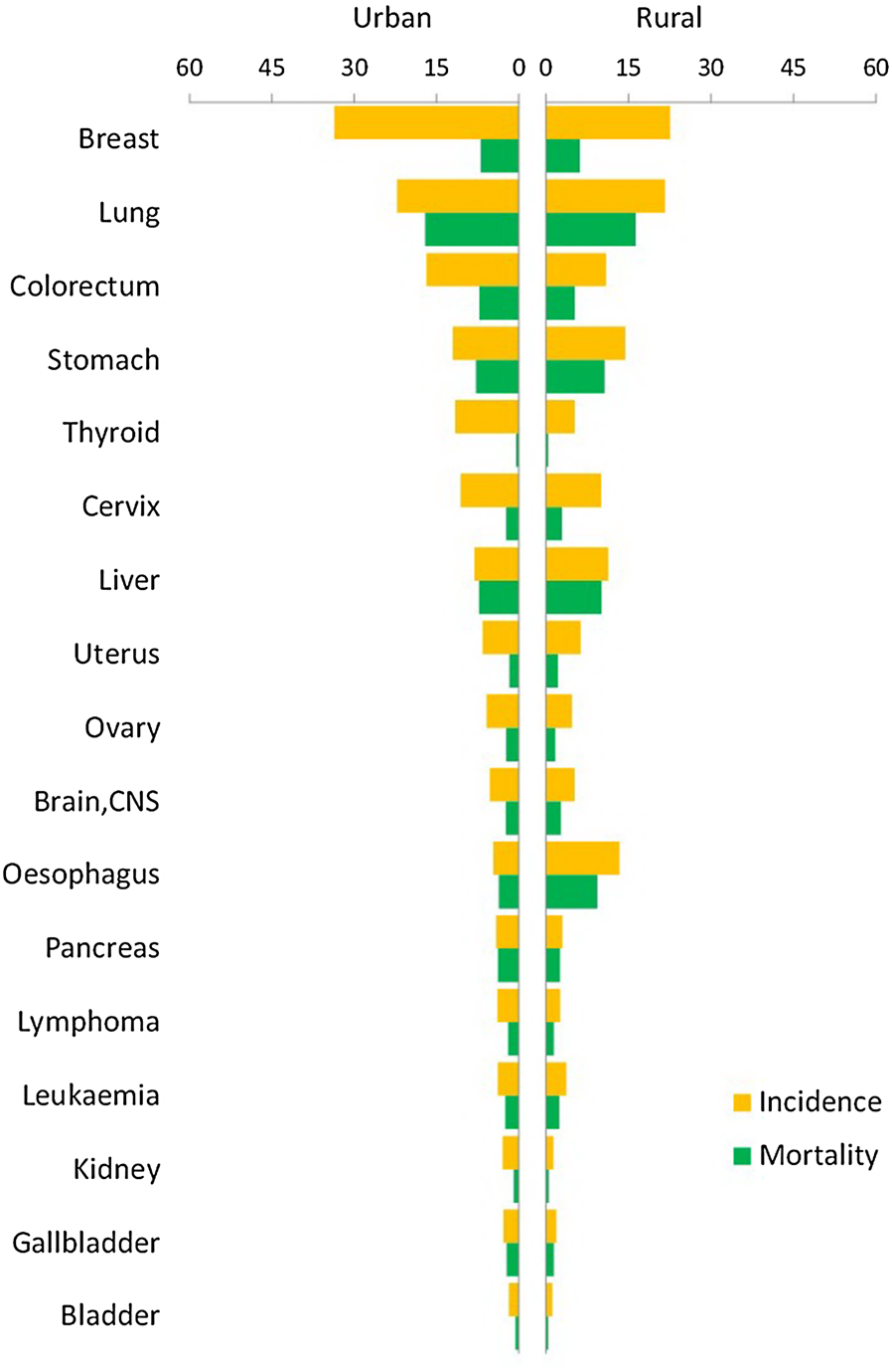
Cancer incidences and mortalities in urban and rural areas for females in China, 2011 (1/1,00,000).

Lung cancer was the most common cancer in all areas, followed by female breast, gastric, liver, and colorectal cancers, with estimated new cases of 651,053, 248,620, 420,489, 355,595, and 310,244 in 2011, respectively. Lung cancer was the most frequently diagnosed cancer in males, followed by gastric, liver, esophageal, and colorectal cancers; breast cancer was the most common cancer in females, followed by lung, colorectal, gastric, and liver cancers (Table 3). The 10 most common cancers accounted for approximately 75% of all cancer cases (Figure 1). In urban areas, lung cancer was the most frequently diagnosed cancer (3,41,543 estimated new cases with an incidence of 49.44/1,00,000), followed by female breast, colorectal, gastric, and liver cancer; in rural areas, lung cancer was the most frequently diagnosed cancer (3,09,510 estimated new cases with an incidence of 47.14/1,00,000), followed by gastric, esophageal, liver, and colorectal cancers (Figures 2, 3).

**Table 3:** Ten cancers with the highest incidences in China, 2011

Rank	Males					Females				
	Site	Cases	Incidence (1/10^5^)	Proportion (%)	ASRIC (1/10^5^)	Site	Cases	Incidence (1/10^5^)	Proportion (%)	ASRIC (1/10^5^)
1	Lung	441,364	63.90	23.01	48.44	Breast	248,620	37.86	17.10	28.51
2	Stomach	296,419	42.92	15.45	32.62	Lung	209,689	31.93	14.43	21.93
3	Liver	264,635	38.32	13.79	29.30	Colorectum	131,840	20.08	9.07	14.02
4	Esophagus	205,560	29.76	10.71	22.47	Stomach	124,070	18.89	8.54	13.21
5	Colorectum	178,404	25.83	9.30	19.70	Liver	90,960	13.85	6.26	9.64
6	Bladder	53,074	7.68	2.77	5.82	Cervix	87,982	13.40	6.05	10.40
7	Prostate	49,007	7.10	2.55	5.33	Esophagus	85,678	13.05	5.89	8.85
8	Pancreas	45,385	6.57	2.37	4.99	Thyroid	67,788	10.32	4.66	8.70
9	Brain, CNS	43,289	6.27	2.26	5.22	Uterus	57,709	8.79	3.97	6.46
10	Lymphoma	41,298	5.98	2.15	4.80	Ovary	45,233	6.89	3.11	5.35

*CNS* central nervous system. Other abbreviations as in Table 1.

**Figure 4:**
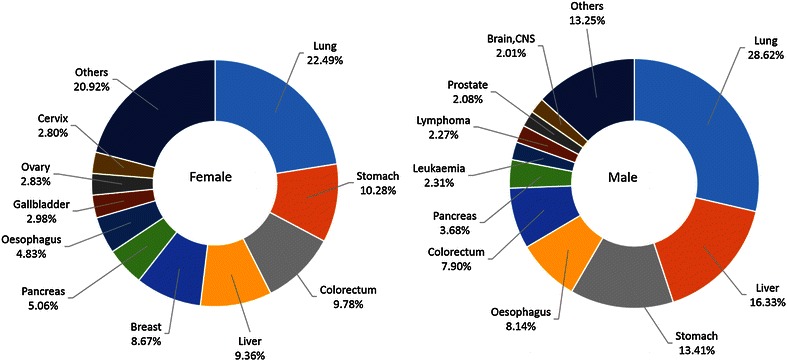
Ten cancers with the highest mortalities in urban areas of China, 2011.

Lung cancer was the leading cause of death in China, followed by liver, gastric, esophageal, and colorectal cancers, with estimated deaths of 529,153, 322,417, 297,496, 218,957, and 149,722, respectively. In males, lung cancer was the leading cause of death, followed by liver, gastric, esophageal, and colorectal cancers; in females, lung cancer was also the leading cause of death, followed by gastric, liver, esophageal, and colorectal cancers (Table 4). The top 10 leading causes of cancer death accounted for approximately 80% of all cancer deaths (Figure 4).

**Table 4:** Ten cancers with the highest mortalities in China, 2011

Rank	Males					Females				
	Site	Deaths	Mortality (1/10^5^)	Proportion (%)	ASRMC (1/10^5^)	Site	Deaths	Mortality (1/10^5^)	Proportion (%)	ASRMC (1/10^5^)
1	Lung	364,432	52.76	27.08	39.94	Lung	164,721	25.08	21.47	16.68
2	Liver	239,218	34.64	17.77	26.38	Stomach	90,792	13.83	11.84	9.21
3	Stomach	206,704	29.93	15.36	22.69	Liver	83,199	12.67	10.85	8.61
4	Esophagus	154,587	22.38	11.48	16.86	Esophagus	64,371	9.80	8.39	6.38
5	Colorectum	86,427	12.51	6.42	9.40	Colorectum	63,295	9.64	8.25	6.26
6	Pancreas	40,580	5.88	3.01	4.43	Breast	60,473	9.21	7.88	6.57
7	Brain, CNS	28,542	4.13	2.12	3.35	Pancreas	32,143	4.89	4.19	3.21
8	Leukemia	27,907	4.04	2.07	3.46	Cervix	23,375	3.56	3.05	2.59
9	Lymphoma	25,066	3.63	1.86	2.84	Brain, CNS	22,234	3.39	2.90	2.54
10	Bladder	20,949	3.03	1.56	2.23	Leukemia	19,708	3.00	2.57	2.45

Abbreviations as in Tables 2 and 3.

The cancer patterns were different between urban and rural areas and between males and females. The main cancers in rural areas were gastric and esophageal cancers, followed by lung, liver, and colorectal cancers, whereas the main cancer in urban areas was lung cancer, followed by liver, gastric, and colorectal cancers.

## Discussion

In 2014, there were 234 registries in China that submitted cancer registry data to the NCCR. The registered populations covered 16.4% of the Chinese population. Through double evaluations of data quality at the provincial and national levels, 177 registries were qualified for inclusion in the final pooled database, which was used for the evaluation of cancer statistics in 2011. For the cancer statistics in 2010, the national population obtained by the sixth Population Census, which only examined the residential population, was used for the cancer statistics estimations [4]. However, the household population is considered more suitable for this analysis because cancer data are based on household registration.

The estimates of cancer incident cases and cancer deaths reasonably increased compared to those in previous years [5, 6], probably because of the aging population and the increasing trends of cancer incidence and mortality. Urban areas in China had a higher cancer incidence and lower cancer mortality than rural areas, and the cancer patterns were quite different between urban and rural areas [2, 3]. A recent study showed that these differences were decreased [7, 8]. Lung cancer is now the most common cancer in rural areas instead of gastric cancer, and this may be due to the high prevalence of tobacco smoking [9] and air pollution [10]. Liver cancer is still one of the most common cancers and one of the leading causes of cancer death in China [11, 12]. A current study showed that rural China had a lower 5-year relative survival rate than urban China [13, 14]. This disparity may be due to limited medical resources, the low number of cancer care centers and the greater number of patients with late-stage cancers in rural areas. The government should be aware of the differences in cancer statistics between urban and rural areas and implement effective measures to bridge the gap.

In 2014, extra funds were provided to the national program to enhance follow-up and survival analyses, which are still lacking in most registries. The registration data should become more valuable and play an important role in cancer control. The National Cancer Center just started a Cancer Atlas Project, which will provide useful information about the geographic distribution of cancers to allow the National Cancer Control Program to be more efficient.
